# Promotion of maize seedling growth by *Enterobacter asburiae* PW2 under salt stress

**DOI:** 10.3389/fpls.2026.1823368

**Published:** 2026-05-04

**Authors:** Mengyuan Wen, Xiu Zhang, Guoping Yang, Xuexian Zhang, Jingyu Li, Fengxia Yu, Haorong Li, Liming Lu, Lankun Long

**Affiliations:** 1 College of Biological Science and Engineering, North Minzu University, Ningxia Key Laboratory for the Development and Application of Microbial Resources in Extreme Environments, Yinchuan, Ningxia, China; 2 Northeast Institute of Geography and Agroecology, Chinese Academy of Sciences, Changchun, Jilin, China

**Keywords:** *Enterobacter asburiae*, maize, osmotic adjustment substances, plant growth-promoting rhizobacteria, salt stress

## Abstract

Plant growth-promoting rhizobacteria (PGPR) are beneficial bacteria that directly or indirectly enhance plant growth, crop yields and stress tolerance. In this study, the maize variety ‘Ningdan 33’ was used as the experimental material. Strain PW2, isolated from salinized soil in Yinchuan, Ningxia, was identified as *Enterobacter asburiae* based on morphological, physiological, and biochemical characteristics as well as 16S rRNA gene sequencing. Pot experiments demonstrated its potential for salt tolerance and growth promotion in maize. The strain can grow in environments with 2.0%–10.0% NaCl and at pH 8.0–11.0, while exhibiting multiple plant growth-promoting traits, such as nitrogen fixation, potassium solubilization, siderophore production, ACC deaminase activity, and IAA synthesis. Under salt stress conditions, inoculation with PW2 significantly promoted maize seed germination, seedling growth, and root system development. Compared to the control group, inoculation with PW2 suspension significantly increased plant height, root length, nitrogen content, and the fresh and dry weights of both shoots and roots. Furthermore, PW2 inoculation significantly enhanced antioxidant enzyme activities in maize leaves and roots. Specifically, the activities of POD, SOD, and CAT in leaves increased by 92.75% (*p* = 4.0×10^-7^), 21.79% (*p* = 0.008), and 17.28% (*p* = 0.010), respectively, while those in roots increased by 54.32% (*p* = 2.0×10^-6^), 19.36% (*p* = 0.041), and 37.80% (*p* = 0.002), respectively. Conversely, the MDA content in leaves and roots decreased by 19.53% (*p* = 0.0058) and 32.91% (*p =* 0.006), respectively. Additionally, the contents of soluble sugar, soluble protein, and proline in both leaves and roots increased significantly. Collectively, these results indicate that strain PW2 effectively mitigates oxidative damage caused by salt stress through enhanced antioxidant defense and improved osmotic regulation, thereby promoting maize growth.

## 1 Introduction

Maize (*Zea mays* L.) is a cornerstone cereal crop vital for global food security, livestock feed, and bioenergy production (Kubi et al., 2021). However, its moderate sensitivity to salt stress poses a significant challenge: excessive salt accumulation suppresses seedling growth, disrupts metabolic and hormonal signaling, and ultimately reduces yield (Zakavi et al., 2022). With over 50% of global arable land projected to face salinization by 2050, and China alone harboring 211 million hectares of salt-affected soils (Li et al., 2024), addressing salt stress is critical for sustaining maize production in saline-alkali environments (Maimaiti et al., 2025).

Plant Growth-Promoting Rhizobacteria (PGPR) offer a promising solution by establishing intimate interactions with plant roots and modulating physiological and biochemical networks (Al-Turki et al., 2023; Iqbal et al., 2024). PGPR enhance salt tolerance through multiple mechanisms: they secrete indole-3-acetic acid (IAA) to optimize root architecture, solubilize phosphorus to improve nutrient uptake, and produce exopolysaccharides (EPS) to chelate Na^+^ and stabilize the rhizosphere (Lu et al., 2024). At the cellular level, PGPR activate antioxidant enzymes (SOD, POD, CAT) (Kumar et al., 2021) to mitigate reactive oxygen species (ROS) damage (Zaki et al., 2025) and synthesize 1-aminocyclopropane-1-carboxylate (ACC) deaminase to reduce ethylene-induced stress (Wang et al., 2024). Additionally, they regulate ion transporters to maintain Na^+^/K^+^ homeostasis (Siddika et al., 2024) and modulate the Salt Overly Sensitive (SOS) and abscisic acid (ABA) pathways, upregulating stress-responsive genes like *APX* (Wang et al., 2025). These synergistic effects stabilize photosynthesis and biomass accumulation under salt stress.

While PGPR-mediated salt tolerance is well-documented (Ning et al., 2024a), the role of *Enterobacter asburiae* in maize adaptation to saline-alkali conditions remains understudied. Here, we hypothesize that inoculation with *E. asburiae* PW2 enhances maize salt tolerance by promoting rhizosphere colonization, triggering hormonal signaling, activating antioxidant defenses, and maintaining ion homeostasis. This study aims to elucidate the mechanistic basis of *E. asburiae*-mediated salt tolerance in maize, providing a novel microbial resource and theoretical framework for bioremediation of saline-alkali agroecosystems.

## 2 Materials and methods

### 2.1 Isolation of salt-tolerant strains and preparation of bacterial suspension

Soil samples were collected in 2025 from the surface layer (10–20 cm) of typical saline-alkali agricultural fields at the foot of Helan Mountain in the Yinchuan region of Ningxia (38°30′ N, 106°00′ E). A total of five samples were collected using the five-point sampling method, with approximately 1 kg of soil per sample. The samples were stored at 4 °C for the isolation and screening of salt-tolerant plant growth-promoting bacteria. A total of 19 bacterial isolates were obtained from the soil samples. The maize variety used was the uncoated ‘Ningdan 33’, and the seeds were purchased from Ningxia Runfeng Seed Co., Ltd (Runfeng Seed Co., Ltd., Ningxia, China).

To isolate PGPR, soil samples were processed via the gradient dilution spread plate method. Specifically, a 10 g sample was suspended in 90 mL of sterile water in a sterilized flask and agitated (180 rpm, 30 °C, 30 min) before resting for 3–5 min. The resulting supernatant underwent serial dilution, and 100 μL from each gradient was inoculated onto LB agar plates. Following incubation (28 °C, 18–24 h), isolates were selected according to morphological traits such as size, shape, and color. Purification was achieved using the streak plate method on Luria–Bertani (LB) agar (prepared in-house with 10 g/L tryptone, 10 g/L NaCl, 5 g/L yeast extract, and 20 g/L agar powder; all components purchased from Beijing Aoboxing Bio-Technology Co., Ltd., Beijing, China), with each step repeated in triplicate. Morphological uniformity served as the criterion for purity. Ultimately, purified strains were cultured in LB broth for 16 h, combined with sterile 60% glycerol (Xuzhou Tianhong Chemical Co., Ltd., Xuzhou, China) (1:1 ratio), and archived at −80 °C.

To prepare the bacterial suspension, purified strains were first grown in LB broth at 30 °C and 180 rpm for 16 h. A 1% (v/v) aliquot of this culture was then transferred to fresh LB broth and incubated for an additional 16 h. Cells were harvested by centrifugation at 12,000×g (Hunan Xiangyi Group, Changsha, China) for 10 min, and the supernatant was removed. The pellet was washed and resuspended in sterile water, then standardized to a concentration of 1.0×10^8^ CFU/mL.

### 2.2 Screening of maize growth-promoting bacteria under salt stress

‘Ningdan 33’ maize seeds that were uniform in size and shape were selected for surface sterilization. The process involved a 10−15 s treatment with 75% ethanol, followed by a 15-min immersion in 6% sodium hypochlorite (Shanghai Guangnuo Chemical Technology Co., Ltd., Shanghai, China). Subsequently, the seeds were rinsed 3–5 times with sterile water to clear away any remaining disinfectant. After a low-temperature treatment at 4 °C for 3–5 h, the seeds were sown on water agar plates. Germination was carried out in the dark at 28 °C and 35% relative humidity for a period of 2 days.

Maize seedlings with consistent growth status were selected and transferred into filter-paper-lined Petri dishes. Three treatments were established: CK0 (no-salt control, Hoagland solution only), CK1 (salt stress control, Hoagland solution + 150 mmol/L NaCl), and the inoculation group (root dipping in bacterial suspension followed by irrigation with Hoagland solution + 150 mmol/L NaCl). Each treatment was performed in triplicate, with four seedlings per dish receiving 10 mL of the respective solution. The dishes were incubated in a light chamber (Shanghai Jinwen Instrument and Equipment Co., Ltd., Shanghai, China) set to simulate mild summer conditions. After three days, the seedlings were measured to determine length changes for analysis.

After surface disinfection with 75% alcohol, tissue culture bottles (Guizhou Hechuang Packaging Co., Ltd., Guizhou, China) were filled with 450 g of sterile vermiculite (Chifu Flagship Store, Taobao.com, China). The experiment comprised five groups to screen for salt stress intensity: a control (CK0, Hoagland solution) and four NaCl treatments (50, 100, 150, and 200 mmol/L NaCl in Hoagland solution). Seeds germinated in the dark for three days were transferred into the vermiculite (~1 cm depth) at a density of 4 seeds per bottle, with three bottles per group. Each bottle was irrigated with 80 mL of the designated solution. Cultivation occurred in a plant incubator set to mimic mild summer conditions, with sterile water (20 mL) added every 3–4 days. After 14 days, growth conditions were evaluated to determine the optimal salinity level for subsequent studies.

To confirm the plant growth-promoting effects observed in the primary screening, a follow-up experiment was conducted using tissue culture flasks. The experimental design included four groups: CK0 (non-saline control), CK1 (100 mmol/L NaCl salt stress control), P0 (bacterial inoculation without salt), and P1 (bacterial inoculation with 100 mmol/L NaCl). All groups were performed in triplicate. During planting, maize sprouts in the inoculation groups were treated with 200 µL of bacterial suspension (1.0×10^8^ CFU/mL) dropped directly onto each germinated seed before being buried under 1 cm of sterile vermiculite. Four seeds were planted per flask, and 80 mL of the designated nutrient solution was added. The flasks were maintained under mild summer climate simulation, with sterile water replenishment every 3–4 days. Following 14 days of growth, morphological indices (plant height and root length) were recorded to screen for strains that effectively mitigated salt stress.

### 2.3 Determination of growth-promoting ability and salt-alkali tolerance of the strains

Strain PW2, which exhibited the highest plant height and root length increases among all candidates in the secondary screening, was purified by streak plating and then subjected to a series of experiments to characterize its growth-promoting mechanisms. Nitrogen fixation was assessed using Ashby medium, siderophore production was identified via the CAS assay, and phosphate solubilization was evaluated using Mengjinna organic phosphorus medium and PKO inorganic phosphorus medium. These three media were all purchased from Hope Bio-Technology Co., Ltd. (Qingdao, China). Furthermore, potassium solubilization was examined using silicate bacteria medium (Schwyn and Neilands, 1987; Glick, 2012). Furthermore, ACC deaminase activity was determined by inoculating the strain into ADF and DF liquid media for 72 h; the OD_600_ was measured using a full-wavelength microplate reader, and the strain was considered positive for ACC deaminase activity if the OD_600_ in the ADF medium was higher than that in the DF medium (Beijing Regen Biotechnology Co., Ltd., Beijing, China) (Penrose and Glick, 2003), and IAA production was measured using the Salkowski colorimetric method with reagents from Fuzhou Flyclean Biotechnology Co., Ltd. (Fuzhou, China) (Glickmann and Dessaux, 1995).

The NaCl tolerance of the strain was evaluated as follows: NaCl (Fuchen Chemical Reagent Co., Ltd., Tianjin, China) was added to LB liquid medium to achieve final concentrations (w/v) of 2%, 4%, 6%, 8%, and 10%. The pH was adjusted to 7.0, followed by sterilization at 121 °C for 20 min. The medium was then inoculated with 1% (v/v) of an 18-h preculture of strain PW2 and incubated at 37 °C with shaking at 150 r/min for 18 h. Growth was quantified by measuring the absorbance at OD_600_ using a full-wavelength microplate reader (Detie Laboratory Equipment Co., Ltd., Nanjing, China) (Mishra et al., 2021).

To assess the alkaline tolerance of the strain, LB liquid media were adjusted to pH values of 8, 9, 10, 11, and 12 using 1 mol/L NaOH (Fuchen Chemical Reagent Co., Ltd., Tianjin, China). After sterilization (121 °C, 20 min), the media were inoculated with 1% (v/v) of an 18-h culture and incubated under the same conditions (37 °C, 150 r/min, 18 h). Growth was monitored by measuring the OD_600_ to identify the suitable alkaline range for strain PW2 (Radhakrishnan et al., 2017).

### 2.4 Physiological, biochemical, and molecular identification of the strain

To characterize strain PW2 and assess its safety, various physiological and biochemical tests were performed. Colony morphology (size, shape, and color) was observed on streak plates, and Gram staining was conducted. Standard biochemical tests—including methyl red (MR), Voges-Proskauer (VP), catalase activity, starch hydrolysis, gelatin liquefaction, ammonia production, and citrate utilization—were carried out according to *Bergey’s Manual of Determinative Bacteriology* (Holt et al., 1994), using commercial reagent kits from Hope Bio-Technology Co., Ltd. (Qingdao, China). Additionally, hemolytic activity was evaluated on Columbia blood agar (Becton Dickinson, Franklin Lakes, NJ, USA) plates as a preliminary biosafety assessment.

For molecular identification based on the 16S rRNA gene, genomic DNA of strain PW2 was extracted using a Bacterial Genomic DNA Extraction Kit, and the gene was amplified via PCR with the universal bacterial primers 27F (5′-AGAGTTTGATCMTGGCTCAG-3′) and 1492R (5′-GGTTACCTTGTTACGACTT-3′) (Sangon Biotech Co., Ltd., Shanghai, China).

The 25 μL PCR reaction mixture contained 12.5 μL of 2× Phanta Max Master Mix, 1 μL of template DNA, 1 μL of each primer, and 9.5 μL of ddH_2_O. The thermal cycling conditions were as follows: initial denaturation at 95 °C for 5 min; 30 cycles of denaturation at 94 °C for 30 s, annealing at 57 °C for 30 s, and extension at 72 °C for 90 s; and a final extension at 72 °C for 10 min, followed by holding at 4 °C.

The PCR amplicons were verified by agarose gel electrophoresis. Qualified products were sent to Sangon Biotech (Shanghai, China) for bidirectional sequencing. The obtained sequences were subjected to BLAST similarity searches against the NCBI GenBank database to retrieve homologous reference sequences. Multiple sequence alignment was performed using MAFFT, and ambiguous or poorly aligned sites were removed with TrimAl.

A Maximum Likelihood (ML) phylogenetic tree was constructed using MEGA 11 based on the aligned dataset under the GTR+I+G substitution model with 1000 bootstrap replicates.

A Bayesian Inference (BI) tree was inferred using MrBayes under the GTR+I+G model (four gamma categories). Two independent runs of 2,000,000 generations were performed, with sampling every 1000 generations. The first 25% of samples were discarded as burn-in, and convergence was confirmed by PSRF values close to 1.0 and ESS values > 200. A 50% majority-rule consensus tree was generated from the remaining trees.

*Cedecea davisiae* (accession number KF146959.1) was used as the outgroup in both analyses.

### 2.5 Determination of the effects of strain PW2 on maize seed germination indices

Uniform ‘Ningdan 33’ maize seeds were surface-sterilized and pre-treated at a low temperature. Subsequently, four treatment groups were established: CK0 (salt-free Hoagland nutrient solution), CK1 (Hoagland solution containing 100 mmol/L NaCl), P0 (salt-free Hoagland solution + PW2 cell suspension), and P1 (Hoagland solution containing 100 mmol/L NaCl + PW2 cell suspension). The seeds were arranged on double-layer filter paper in Petri dishes (15 seeds per dish). One drop of PW2 cell suspension was added to the P0 and P1 groups, whereas the CK0 and CK1 groups remained uninoculated. Then, 15 mL of the corresponding nutrient solution was added to each dish. Each treatment was conducted in triplicate.The dishes were incubated in a plant growth chamber for 7 d, with 10 mL of sterile water supplemented to each dish on day 3. The number of seeds with a plumule length exceeding 2 mm was recorded daily to calculate the germination rate, germination energy, germination index, and vigor index. After 7 d of cultivation, 10 uniformly growing seedlings were randomly selected from each treatment to measure their plumule and radicle lengths, and their fresh weights were recorded. The seedlings were then fixed at 105 °C for 30 min in an oven (Shanghai Hengke Scientific Instrument Co., Ltd., Shanghai, China) to deactivate enzymes, followed by drying at 80 °C to a constant weight to determine the dry weight (Hudu et al., 2025).


$$Germination\;Percentage\;\left(\%\right)\;=\;Gt=7/N\;\times \;100$$

$$Germination\;Energy\;\left(\%\right)\;=\;Gt=3/N\;\times \;100$$

$$Germination\;Index\;=\;\sum \left(Gt/Dt\right)$$

$$Mean\;Germination\;Time\;\left(d\right)\;=\;\left(\sum \left(Gt\;-\;Gt-1\right)\;\times \;Dt\right)/Gt=7$$

Where: Gt=7 is the number of germinated seeds on day 7, N is the number of tested seeds; Gt=3 is the number of germinated seeds on day 3; Gt is the number of germinated seeds on day t, and Dt is the corresponding germination time (d) (Prerna et al., 2021; Rahbari and Madandoust, 2024; Fu et al., 2025).

### 2.6 Determination of growth parameters and physiological indices of maize seedlings treated with strain PW2

A hydroponic validation experiment in tissue culture bottles was conducted using the rescreened strain PW2. Four treatment groups were established: CK0 (Hoagland nutrient solution, no salt stress), CK1 (Hoagland solution + 100 mmol/L NaCl, salt stress control), P0 (Hoagland solution + PW2 strain), and P1 (Hoagland solution + 100 mmol/L NaCl + PW2 strain). Each treatment was performed in triplicate. After 14 days of cultivation in a plant growth chamber, five seedlings were randomly selected from each replicate for parameter evaluation. Plant height and root length were measured using a ruler (accurate to 1 mm), while stem diameter was measured with a vernier caliper (Shanghai Measuring & Cutting Tool Works Co., Ltd., Shanghai, China). The relative chlorophyll content (SPAD value) and nitrogen content of the leaves were determined using a handheld chlorophyll meter (Beijing Zhongke Weihe Technology Development Co., Ltd., Beijing, China). Following the measurement of shoot and root fresh weights using an electronic balance (Shanghai Yixi Instrument Equipment Co., Ltd., Shanghai, China), the samples were fixed at 105 °C for 30 min to deactivate enzymes, and then dried at 80 °C to a constant weight to determine the dry biomass. Furthermore, complete root system images were acquired using a root scanner (Shanghai Zhongjing Technology Co., Ltd., Shanghai, China), and root morphological parameters—including total root length, total root volume, total root surface area, and root tip number—were analyzed using dedicated root analysis software (Sichuan Ruijinte Technology Co., Ltd., Sichuan, China).

Physiological parameters, including soluble sugar, soluble protein, proline, dehydrogenase, extracted chlorophyll, malondialdehyde (MDA), catalase (CAT), peroxidase (POD), and superoxide dismutase (SOD), were determined using commercially available assay kits from Comin Biotechnology Co., Ltd. (Suzhou, Jiangsu, China) in strict accordance with the manufacturer’s instructions. All measurements were performed with three biological replicates. For each biological replicate, leaf samples were pooled from six individual plants. Three technical replicates were measured per biological replicate, and the results were expressed as mean ± standard deviation (*SD*, n = 3) (Yin et al., 2018; Wang et al., 2019; Xiao et al., 2023).

Soluble sugar was determined using a commercial kit (KT-1-Y, Comin, China). Fresh leaves (0.1–0.2 g) were homogenized in 1 mL distilled water, incubated in a capped tube at 95 °C for 10 min, and centrifuged at 8,000×g for 10 min. An aliquot of 0.1 mL of the supernatant was mixed with 0.9 mL distilled water (10-fold dilution). The working solution was prepared by dissolving reagent 1 in 2.5 mL of reagent 2. For the assay, 40 μL of the diluted sample, 40 μL of working solution, and 200 μL of concentrated H_2_SO_4_ were mixed in a tube (the blank used 80 μL of water instead of the sample). After capping, the tubes were incubated at 95 °C for 10 min. Then, 200 μL of the reaction mixture was transferred to a 96-well plate, and the absorbance was read at 620 nm using a microplate reader. Δ*A* = *A*_sample − *A*_blank. Soluble sugar content (mg/g FW) was calculated as: Content = 1.17 × (Δ*A* + 0.07) ÷ W, where W is the fresh weight (g) of the sample.

Soluble protein was determined using a BCA kit (Comin, China). Fresh leaves (0.1 g) were homogenized in 1 mL distilled water on ice, centrifuged at 12,000×g for 10 min at 4 °C, and the supernatant was collected. The working solution was preheated at 60 °C for 30 min. Then, 200 μL of distilled water (blank), standard solution, or sample was mixed with 200 μL of working solution, incubated at 60 °C for 30 min. An aliquot of 200 μL of the reaction mixture was transferred to a 96-well plate, and the absorbance was read at 562 nm (*A*_blank, *A*_standard, *A*_sample). Protein content (mg/g FW) = C_standard × (*A*_sample − *A*_blank) ÷ (*A*_standard − *A*_blank) × 1 mL ÷ W.

Proline was determined using a kit (Comin, China). Fresh leaves (0.1 g) were homogenized in 1 mL extraction buffer, incubated with shaking at 90 °C for 10 min, centrifuged at 10,000×g for 10 min at 25 °C, and the supernatant was cooled. For the assay, 250 μL of the sample was mixed with 250 μL of reagent 1 and 250 μL of reagent 2 in a capped tube, incubated in a boiling water bath for 30 min (shaken every 10 min). After cooling, 500 μL of reagent 3 was added, shaken for 30 s, and allowed to stand. Then, 200 μL of the upper layer was transferred to a 96-well plate, and the absorbance was read at 520 nm (*A*). Proline content (μg/g FW) = 38.4 × (*A* + 0.0021) ÷ W.

Dehydrogenase was determined using a kit (Comin, China). Fresh root samples (0.1 g) were washed, placed in a 10 mL centrifuge tube, and mixed with 0.4 mL of reagent 1 and 0.4 mL of reagent 2. After thorough mixing, the tube was incubated at 37 °C in the dark for 2 h. The roots were then removed, surface moisture was blotted dry, and transferred to a new tube containing 2 mL of methanol, followed by incubation at 40 °C for 2 h. An aliquot of 200 μL of the supernatant was transferred to a 96-well plate, and the absorbance was read at 485 nm (*A*). Δ*A* = *A*_sample − *A*_blank. Dehydrogenase activity (μg/(g·min)) = 0.789 × (Δ*A* + 0.0312) ÷ W.

Chlorophyll was determined using a kit (Comin, China). Fresh leaves (0.1 g) were deveined, cut into pieces, and washed. The sample was ground with 1 mL distilled water and approximately 50 mg of reagent 1 (powder) under dark or low-light conditions, then transferred to a 10 mL glass tube. The mortar was rinsed with extraction buffer (distilled water:acetone = 1:4) and the volume was brought to 10 mL. The tube was kept in the dark for 3 h until the tissue residue became completely white. An aliquot of 200 μL of the extract was transferred to a 96-well plate, and the absorbance was read at 645 nm and 663 nm (*A*_645_, *A*_663_) using the extraction buffer as a blank. Total chlorophyll content (mg/g FW) = 0.01 × (20.21 × *A*_645_ + 8.02 × *A*_663_) ÷ W.

Malondialdehyde (MDA) was determined using a kit (Comin, China). Fresh leaves (0.1 g) were homogenized in 1 mL extraction buffer on ice, centrifuged at 8,000×g for 10 min at 4 °C, and the supernatant was kept on ice. Then, 0.3 mL of reagent 1 was mixed with 0.1 mL of the sample in a 1.5 mL tube, capped, and incubated in a 95 °C water bath for 30 min. After cooling on ice, the mixture was centrifuged at 10,000×g for 10 min at 25 °C. An aliquot of 200 μL of the supernatant was transferred to a 96-well plate, and the absorbance was read at 532 nm and 600 nm (*A*_532_, *A*_600_). Δ*A* = *A*_532_ − *A*_600_. MDA content (nmol/g FW) = 51.6 × Δ*A* ÷ W.

Catalase (CAT) was determined using a kit (Comin, China). Fresh leaves (0.1 g) were homogenized in 1 mL extraction buffer on ice, centrifuged at 8,000×g for 10 min at 4 °C, and the supernatant was kept on ice. Reagents were added to EP tubes according to the manufacturer’s instructions (control and sample tubes), mixed, and incubated at 25 °C for exactly 10 min, followed by addition of stop solution. An aliquot of 200 μL of the reaction mixture was transferred to a 96-well plate, and the absorbance was immediately read at 405 nm (*A*_sample, *A*_control). Δ*A* = *A*_control − *A*_sample. CAT activity (μmol/(min·g FW)) = 8.9 × (Δ*A* − 0.0013) ÷ W.

Peroxidase (POD) was determined using a kit (Comin, China). Fresh leaves (0.1 g) were homogenized in 1 mL extraction buffer on ice, centrifuged at 8,000×g for 10 min at 4 °C, and the supernatant was kept on ice. The working solution was prepared according to the manufacturer’s instructions and preheated at 25 °C. In a 96-well plate, 10 μL of sample was mixed with 190 μL of working solution, and the absorbance was recorded at 470 nm at 1 min and 2 min (*A*1, *A*2). Δ*A* = *A*2 − *A*1. POD activity (U/g FW) = 4000 × Δ*A* ÷ W.

Superoxide dismutase (SOD) was determined using a kit (Comin, China). Fresh leaves (0.1 g) were homogenized in 1 mL extraction buffer on ice, centrifuged at 8,000×g for 10 min at 4 °C, and the supernatant was collected and kept on ice. The working solution was prepared according to the manufacturer’s instructions. After mixing thoroughly, the mixture was allowed to stand at room temperature for 30 min, and the absorbance was then read at 450 nm (sample tube and control tube). Inhibition percentage (%) = (*A*_control − *A*_sample)/*A*_control × 100%. SOD activity (U/g FW) = 20 × inhibition percentage ÷ (1 − inhibition percentage) ÷ W.

### 2.7 Statistical data analysis

Biological experiments were independently repeated multiple times to ensure reproducibility. To rigorously handle data from repeated experiments, the mean values of biological replicates within each independent experimental run were calculated first. These ‘experiment-wise means’ were then used as the input data for subsequent statistical analysis to ensure the variance reflected biological reproducibility. The final results are reported as the mean ± standard deviation (*SD*) of these independent experimental repeats. Prior to analysis, the assumptions of normality and homogeneity of variances were confirmed using the Shapiro-Wilk test and Levene’s test, respectively. One-way analysis of variance (ANOVA) was performed using SPSS 26.0, with statistical significance defined as *p < 0.05*. Graphing was carried out using Origin 2025b. Additionally, software utilized for sequence alignment and phylogenetic analysis included MEGA 11 and MrBayes.

## 3 Results

### 3.1 Results of bacterial screening for promoting maize growth under salt stress

In terms of root length, one-way ANOVA and Tukey’s multiple comparison test revealed that the root length in the salt stress group (CK1, 2.425 cm) was significantly reduced compared to the normal control group (CK0, 7.425 cm). Compared with CK1, all eight selected candidate strains (YX6, Y2, Y7, B10, XC3, PW2, YX11, and Q8) significantly increased root length, with an increase ranging from 49.48% to 71.13%. Among them, the root length following treatment with strain Y2 (4.150 cm) was the highest and differed significantly from the other seven strains (indicated by different letters in **Table 1**).

**Table 1:** Preliminary screening results of some strains on maize root length (mean ± *SD*, n=4).

Treatment	Root Length/cm	Treatment	Root Length/cm
CK0	7.425 ± 0.618^a^	Q16	2.050 ± 0.332^g^
CK1	2.425 ± 0.634^fg^	PW2	3.750 ± 0.300^bcd^
YX6	3.900 ± 0.983^bc^	YX11	3.800 ± 0.497^bcd^
XJ21	3.125 ± 0.506^bcdef^	PW6	2.925 ± 0.320^cdefg^
NM6	2.850 ± 0.742^defg^	XJ17	2.075 ± 1.075^g^
NM3	2.900 ± 0.383^cdefg^	SC5	2.925 ± 0.723^cdefg^
B4	2.550 ± 0.412^fg^	QW2	2.675 ± 0.320^efg^
Y2	4.150 ± 0.810^b^	QW5	2.475 ± 0.427^fg^
Y7	3.725 ± 0.978^bcd^	Q8	4.075 ± 0.574^b^
B10	3.625 ± 0.768^bcde^	QY9	3.200 ± 0.337^bcdef^
XC3	3.675 ± 0.263^bcde^		
Overall statistics	*CV* = 43.0% *F* = 2.11 *p* = 0.015		

Preliminary screening results of different strains on maize root length. Values are presented as mean ± standard deviation (*SD*, n=4). Overall statistics: coefficient of variation (*CV*) = 43.0%, *F* value = 2.11, *p* value = 0.015 (one-way ANOVA). Values with the same letter in the same column indicate no significant difference between treatments (Tukey’s test, *p > 0.05*).

**Figure 1:**
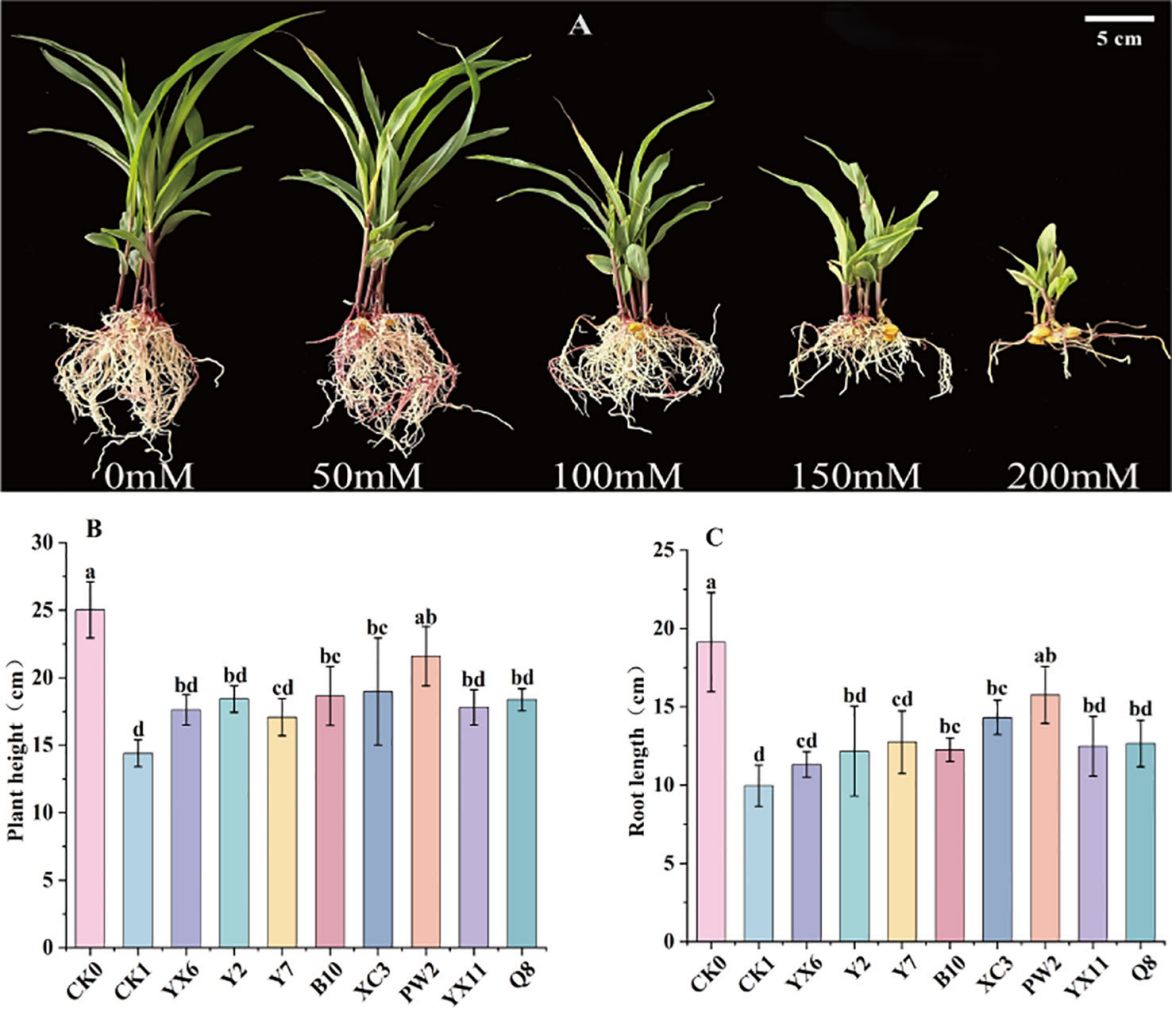
Growth status of maize seedlings **(A)** effects of different treatment groups on maize biomass [**(B)** plant height; **(C)** root length]. CK0, control without salt stress; CK1, control under 100 mmol/L salt stress; P0, inoculation group without salt stress; P1, inoculation group under salt stress. Error bars represent standard deviation (n=4). Different lowercase letters indicate significant differences among treatments at *p < 0.05*.

**Figure 1A** shows that under different salt stress conditions, maize grew normally at 0 mmol/L and 50 mmol/L NaCl concentrations. At 150 mmol/L and 200 mmol/L NaCl concentrations, maize growth was significantly inhibited and the plants were severely damaged. At 100 mmol/L NaCl concentration, maize was inhibited with a moderate stress level. Therefore, 100 mmol/L NaCl was selected as the stress concentration to verify the growth - promoting effect of salt - tolerant and growth - promoting bacteria on maize.

A secondary screening experiment was conducted on the eight candidate strains obtained from the preliminary screening, and the plant height and root length of maize seedlings treated with different strains were determined (**Figures 1B, C**). Statistical analysis indicated that, in terms of plant height, all eight treatment groups exhibited significantly greater plant height compared to the salt stress control group (CK1). Among them, strain PW2 treatment resulted in the highest average plant height, showing an increase of 50.00% compared to CK1 (*p* = 0.00015). Similarly, for root length, all eight treatment groups were significantly longer than CK1, with strain PW2 showing the largest increase of 58.23% (*p* = 0.0006). Based on the above growth parameters, strain PW2 was selected as a salt-tolerant plant growth-promoting bacterium for subsequent experiments.

### 3.2 Determination of growth-promoting and salt-alkali tolerance abilities of the strain

Analysis of the plant growth-promoting characteristics of strain PW2 revealed that the strain possessed multiple capabilities, including nitrogen fixation, potassium solubilization, siderophore production, indole-3-acetic acid (IAA) synthesis, and ACC deaminase activity, but lacked phosphate solubilization capacity (**Table 2**). In terms of quantitative indices, the IAA production was 166.17 ± 4.44 μg/mL (n=3), the siderophore production capacity (D/d) was 1.93 ± 0.23, and the potassium solubilization capacity (D/d) was 1.52 ± 0.08. After 72 h of incubation, the OD_600_ of strain PW2 in ADF medium (0.102 ± 0.010) was significantly higher than that in DF medium (0.065 ± 0.008) (*p* = 0.007), confirming that the strain possessed ACC deaminase activity (Yang et al., 2024). These results confirm that strain PW2 possesses diverse plant growth-promoting traits.

**Table 2:** Growth-promoting characteristics of strain PW2.

Test indicators	Results	Data are shown as mean ± *SD* (n=3)
Nitrogen fixation	+	−
Organophosphorus solubilizing	−	−
Inorganic phosphorus	−	−
Potassium dissolving	+	D/d = 1.52 ± 0.08
Siderophore production	+	D/d =1.93 ± 0.23
ACC deaminase activity	+	OD_600_ (ADF/DF) = 0.102 ± 0.010/0.065 ± 0.008
IAA synthesis	+	166.17 ± 4.44 μg/mL

Data are presented as qualitative (+/–) or quantitative (mean ± *SD*) values. Error bars represent standard deviation (for quantitative data). Siderophore production and potassium solubilization capabilities are expressed as the ratio of halo diameter to colony diameter (D/d). ACC deaminase activity was determined as positive based on growth (OD_600_) in ADF+ACC medium. Nitrogen fixation, potassium solubilization, siderophore production, ACC deaminase activity, and IAA production were positive (+); whereas organic and inorganic phosphate solubilization were negative (–).

**Figure 2:**
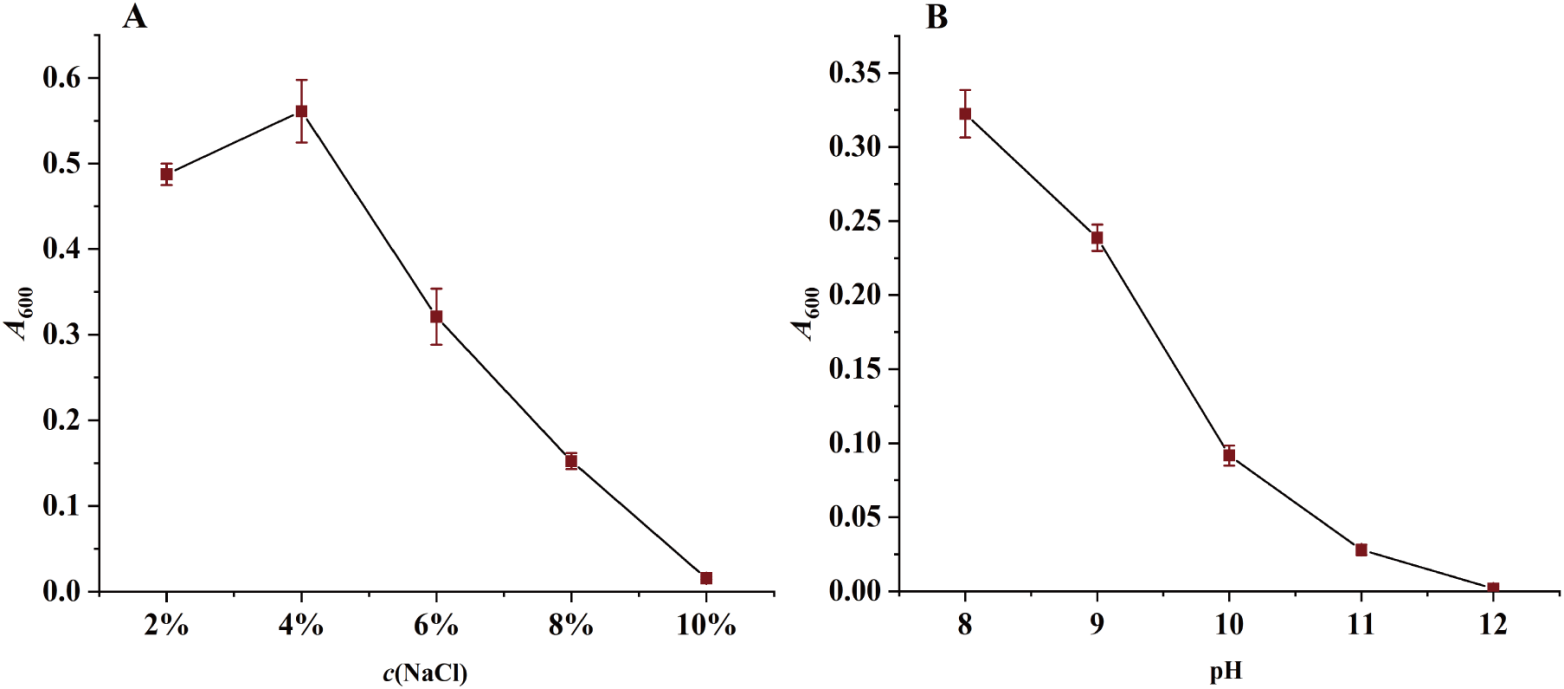
Determination of salt and alkali tolerance of strain PW2. **(A)** Effect of NaCl concentration on the growth of strain PW2, measured by *A*600. **(B)** Effect of pH on the growth of strain PW2, measured by *A*600. Data are presented as mean ± *SD* (n=3).

The determination of the salt and alkali tolerance of strain PW2 showed that the strain was able to grow at NaCl concentrations ranging from 2%–10%, with optimal growth at 4%, followed by a gradual decline (**Figure 2A**). It could grow within the pH range of 8.0–11.0, but no growth occurred at pH 12.0 (**Figure 2B**).

### 3.4 Results of physiological, biochemical, and molecular identification of the strain

**Figure 3:**
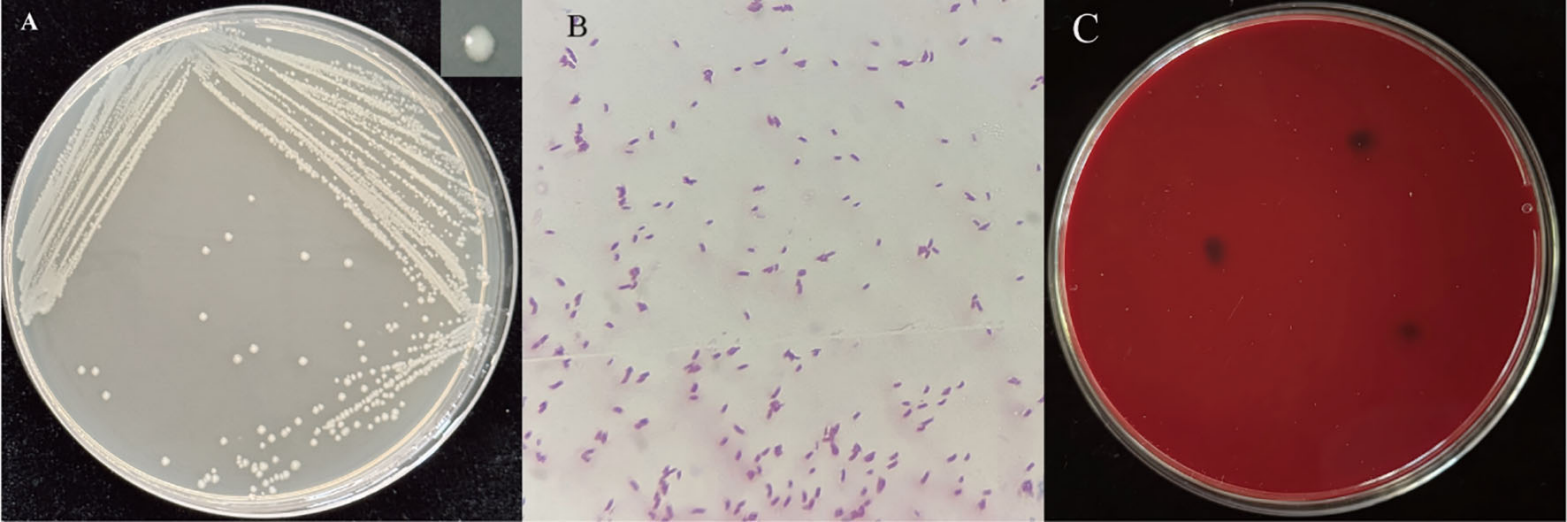
Colony morphology **(A)**, gram staining [**(B)**,100×] and safety test **(C)** of strain PW2.

The colony morphology of strain PW2 was observed after culturing on LB solid medium for 2 days. The colonies were smooth, glossy, and moist, circular and convex with entire edges, opaque, and yellowish-white. The colony diameter was approximately 2–4 mm (**Figure 3A**). Gram staining resulted in a red color, and the cells were short rod-shaped (**Figure 3B**). Columbia blood agar medium was used to evaluate the safety of strain PW2 by testing for hemolysis. No transparent zone appeared, indicating that strain PW2 exhibits no hemolytic activity (**Figure 3C**).

The results of physiological and biochemical tests showed that strain PW2 was Gram-negative. Methyl red was negative, while V-P and catalase were positive. The strain could utilize citrate, produce fluorescent pigments, and hydrolyze starch, but could not hydrolyze gelatin. The ammonia production test was positive (**Table 3**).

**Table 3:** Physiological and biochemical characteristics of the PW2 strain.

Test items	Results
Gram stain	−
Methylic−red test	−
V−P test	+
Contact enzyme	+
Citrate test	+
Fluorescent pigment	+
Hydrolysis of starch	+
Gelatin liquefaction	−
Ammonia production test	+

+: Postive, −: Negative.

**Figure 4:**
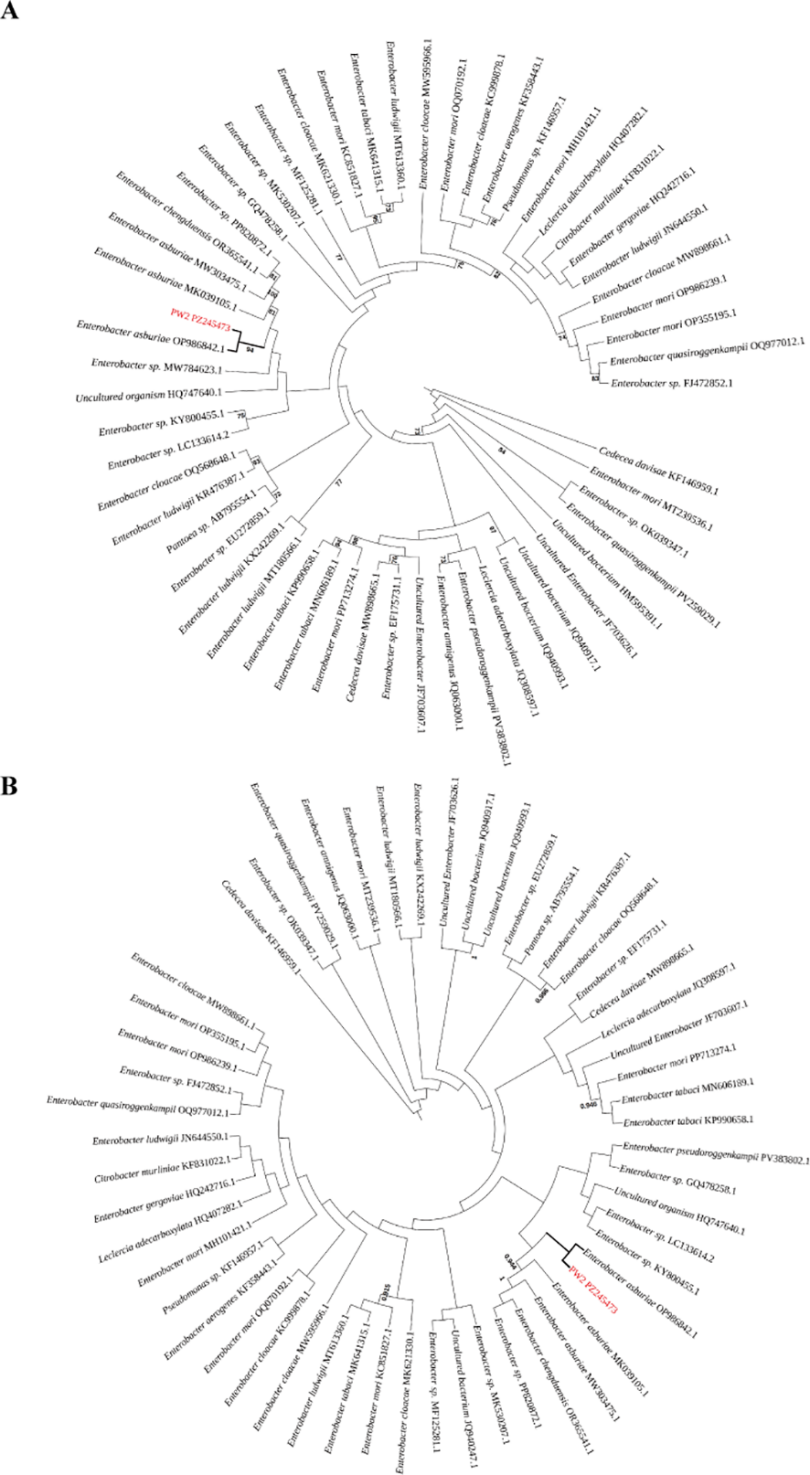
Phylogenetic relationships of strain PW2 and closely related *Enterobacter* species inferred from 16S rRNA gene sequences. **(A)** Maximum Likelihood (ML) tree; **(B)** Bayesian Inference (BI) tree. Both trees were constructed using the GTR+I+G substitution model. Bootstrap values (ML, 1000 replicates) and posterior probabilities (BI) are indicated at the corresponding nodes. Strain PW2 (GenBank accession no. PZ245473) is highlighted in red. *Cedecea davisiae* (KF146959.1) was used as the outgroup.

Strain PW2 was identified as *Enterobacter asburiae* based on morphological, physiological, biochemical characteristics, and 16S rRNA gene phylogenetic analysis. Phylogenetic trees constructed using Maximum Likelihood (ML) and Bayesian Inference (BI) methods (**Figures 4A, B**) revealed that the 16S rRNA gene sequence of PW2 robustly clustered with reference strains of *E. asburiae* (e.g., MK030473.1), forming a well-supported clade with high bootstrap values (ML, >95%) and posterior probabilities (BI, >0.99). This phylogenetic placement strongly corroborated the identification of PW2 as *Enterobacter asburiae*. The 16S rRNA gene sequence was deposited in GenBank under accession number PZ245473, and the strain was deposited at the China Center for Type Culture Collection (CCTCC) under accession number CCTCC M 2026637.

### 3.5 Effect of strain PW2 on maize seed germination under salt stress

As shown in **Table 4**, under 100 mmol/L salt stress conditions, the germination rate of the P0 treatment group was significantly higher than other groups, reaching 88.89%. The germination energy, germination index, and vigor index of the CK0 treatment group were all significantly higher than other groups. All values of the P1 treatment group were significantly higher than those of the CK1 treatment group, indicating that strain PW2 has salt-tolerant and growth-promoting characteristics.

**Table 4:** Maize seed germination and biomass under salt stress (mean ± *SD*, n=10). Error bars represent standard deviation.

Test items Treatment	CK0	CK1	P0	P1	*CV* (%)	*F* value	*p* value
Germination Rate/%	86.67 ± 6.67a	62.22 ± 10.18b	88.89 ± 3.85a	68.89 ± 3.85b	17.20	11.68	0.002
Germination Energy/%	71.11 ± 7.70a	20.00 ± 6.67b	66.67 ± 6.67a	31.11 ± 3.85b	50.16	47.86	<0.001
Germination Index	13.18 ± 1.29a	7.05 ± 1.15b	12.96 ± 0.98a	8.47 ± 0.90b	28.53	24.53	<0.001
Vitality Index	11.90 ± 0.15a	4.00 ± 0.18c	11.70 ± 0.08a	6.38 ± 0.13b	42.00	2466.7	<0.001
Sprout Length/cm	4.47 ± 0.34a	3.25 ± 0.27b	4.73 ± 0.52a	3.65 ± 0.32b	17.53	34.13	<0.001
Root Length/cm	11.39 ± 0.90a	6.65 ± 0.88c	11.07 ± 0.92a	8.03 ± 0.44b	23.45	82.75	<0.001
Fresh Weight/g	0.91 ± 0.10a	0.59 ± 0.072c	0.95 ± 0.12a	0.77 ± 0.10b	21.57	25.55	<0.001
Dry Weight/g	0.38 ± 0.03a	0.33 ± 0.05b	0.35 ± 0.05ab	0.34 ± 0.03ab	13.10	3.43	0.026

Maize seed germination and biomass under salt stress (mean ± *SD*, n=10). *CV* values, *F* values, and *p* values were calculated by one - way ANOVA. Values with the same letter in the same column indicate no significant difference between different groups (*p < 0.05*). CK0, control without salt stress; CK1, control under 100 mmol/L salt stress; P0, inoculation group without salt stress; P1, inoculation group under salt stress. Error bars represent standard deviation (n=10). Different lowercase letters indicate significant differences among treatments at *p < 0.05*.

Under 100 mmol/L salt stress conditions, the stem length of the P0 treatment group was the longest (4.73 cm), the root length of the CK0 treatment group was the longest (11.39 cm), the fresh weight of the P0 treatment group was the highest (0.95 g), and the dry weight of the CK0 treatment group was the highest (0.38 g). Compared with the CK0 treatment group, the P0 treatment group did not show a significant growth-promoting effect on maize seed germination, while the P1 treatment group showed a significant salt-tolerant and growth-promoting effect compared with the CK1 treatment group.

### 3.6 Effect of strain PW2 on maize seedling growth under salt stress

**Figure 5:**
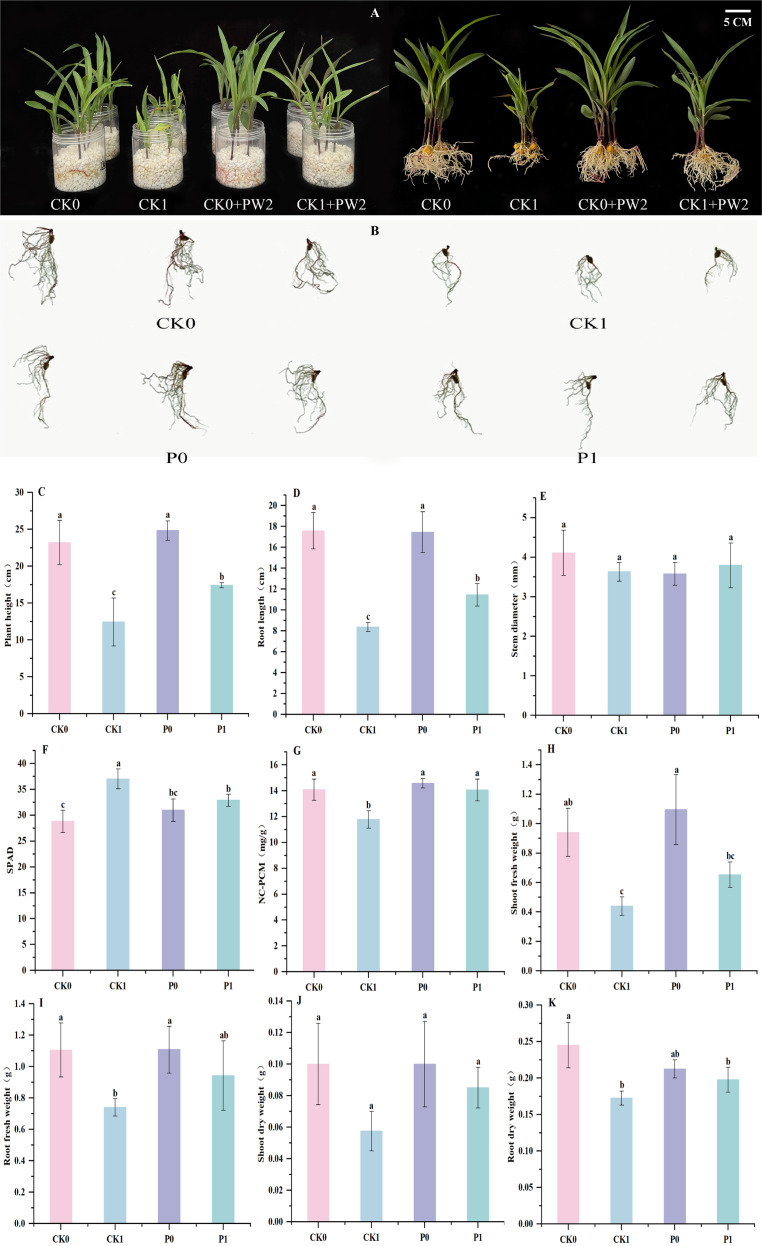
Effects of strain PW2 on morphological parameters of maize seedlings under 100 mmol/L NaCl stress. [**(A)** Growth status; **(B)** Root morphology; **(C)** Plant height; **(D)** Root length; **(E)** Stem diameter; **(F)** SPAD; **(G)** N content; **(H)** Shoot fresh weight; **(I)** Root fresh weight; **(J)** Shoot dry weight; **(K)** Root dry weight]. CK0, control without salt stress; CK1, control under 100 mmol/L salt stress; P0, inoculation group without salt stress; P1, inoculation group under salt stress. Error bars represent standard deviation (n=4). Different lowercase letters indicate significant differences among treatments at *p < 0.05*.

In the hydroponic experiment, the effects of different treatment groups on various growth indicators of maize 14 days after inoculation are shown in **Figure 5A**. Root scanning images (**Figure 5B**) revealed significant differences in maize root morphology under different treatments. The root system in the CK0 group was well-developed, with dense lateral roots and elongated main roots, reflecting good growth under normal conditions. In contrast, the root system in the CK1 group was severely stunted, with sparse lateral roots and short main roots, indicating that salt stress treatment strongly inhibited root morphology. After inoculation with strain PW2, there were no significant differences in lateral root density and main root length between the P0 group and the CK0 group. However, the P1 group showed significant differences compared to the CK1 group, with an increased number of lateral roots and restored main root elongation, indicating that strain PW2 possesses salt-tolerant growth-promoting capabilities.

Under normal conditions (0 mmol/L NaCl), compared to the CK0 treatment group, the maize seedlings in the P0 treatment group showed no significant differences in plant height, root length, stem diameter, SPAD value, nitrogen content, fresh weight, or dry weight, with only a 7% increase in plant height. Under 100 mmol/L NaCl treatment, compared to the CK1 treatment, the P1 treatment significantly improved maize seedling growth. Specifically, plant height increased by 39.76% (17.40 ± 0.34 cm *vs.* 12.45 ± 3.25 cm, *p* = 0.023); root length increased by 36.72% (11.45 ± 1.09 cm *vs.* 8.38 ± 0.44 cm, *p* = 0.002); nitrogen content (NC-PCM) increased by 19.32% (14.05 ± 0.86 mg/g *vs.* 11.78 ± 0.63 mg/g, *p* = 0.005); shoot fresh weight increased by 48.30% (0.65 ± 0.09 g *vs.* 0.44 ± 0.06 g, *p* = 0.007); root fresh weight showed a non-significant increasing trend of 27.36% (0.94 ± 0.21 g *vs.* 0.74 ± 0.06 g, *p* = 0.104); shoot dry weight increased by 47.83% (0.09 ± 0.01 g *vs.* 0.06 ± 0.01 g, *p* = 0.018); and root dry weight increased by 14.49% (0.20 ± 0.02 g *vs.* 0.17 ± 0.01 g, *p* = 0.031). However, neither SPAD value (which exhibited a non-significant decrease of 12.62%) nor stem diameter showed significant changes (**Figures 5C–K**). These results indicate that inoculation with strain PW2 has a salt-tolerant and growth-promoting effect on maize seedlings.

Quantitative measurements of total root length, total root surface area, total root volume, and root tip number (**Table 5**) further validated the phenotypic differences. For total root length, the CK1 group showed a significant reduction to 277.66 cm, while the CK0 group was 539.44 cm; the P0 group had a slight increase to 552.89 cm with no significant difference from the CK0 group; the P1 group recovered to 451.07 cm, significantly higher than the CK1 group. Regarding total root surface area, the CK0 group was significantly higher than other groups at 773.51 cm², the CK1 group decreased to 430.63 cm², the P0 group was 692.24 cm², and the P1 group was 576.34 cm², with significant differences among groups and a gradient change. For total root volume, the CK0 group (217.26 ± 25.97 cm³) and P0 group (227.41 ± 34.64 cm³) showed no significant difference between them but were both significantly higher than the CK1 group (128.91 cm³); the P1 group had a total root volume of 149.88 cm³, which recovered compared to the CK1 group but was still significantly lower than the CK0 and P0 groups. The root tip number of the CK0 group (44.67) and P0 group (47.67) showed no significant difference and were significantly higher than the CK1 group (23.67); the P1 group had 35.33 root tips, significantly higher than the CK1 group but lower than the CK0 and P0 groups.

**Table 5:** Effects of strain PW2 on root indices of maize seedlings under 100 mmol/L NaCl stress.

Treatment	Total root length (cm)	Total root surface area (cm²)	Total root volume (cm³)	Number of root tips
CK0	539.44 ± 42.10ab	773.51 ± 83.73a	217.26 ± 25.97a	44.67 ± 3.06a
CK1	277.66 ± 80.01c	430.63 ± 34.21c	128.91 ± 26.74b	23.67 ± 5.51c
P0	552.89 ± 23.73a	692.24 ± 54.26ab	227.41 ± 34.64a	47.67 ± 2.08a
P1	451.07 ± 39.50b	576.34 ± 54.81b	149.88 ± 34.63b	35.33 ± 3.06b
*CV* (%)	26.94	23.28	28.41	27.10
*F* value	18.78	18.84	7.55	26.48
*p* value	<0.001	<0.001	0.010	<0.001

Effects of strain PW2 on root indices of maize seedlings under 100 mmol/L NaCl stress. Values are presented as mean ± standard deviation (*SD*, n=3). Coefficient of variation (*CV*, %), *F* value, and *p* value are shown in the table. Values with the same letter in the same column indicate no significant difference between treatments (Tukey’s test, *p < 0.05*).

Error bars represent standard deviation (mean ± *SD*, n=3).

### 3.7 Effect of strain PW2 on physiological indices of maize seedlings under salt stress

**Figure 6:**
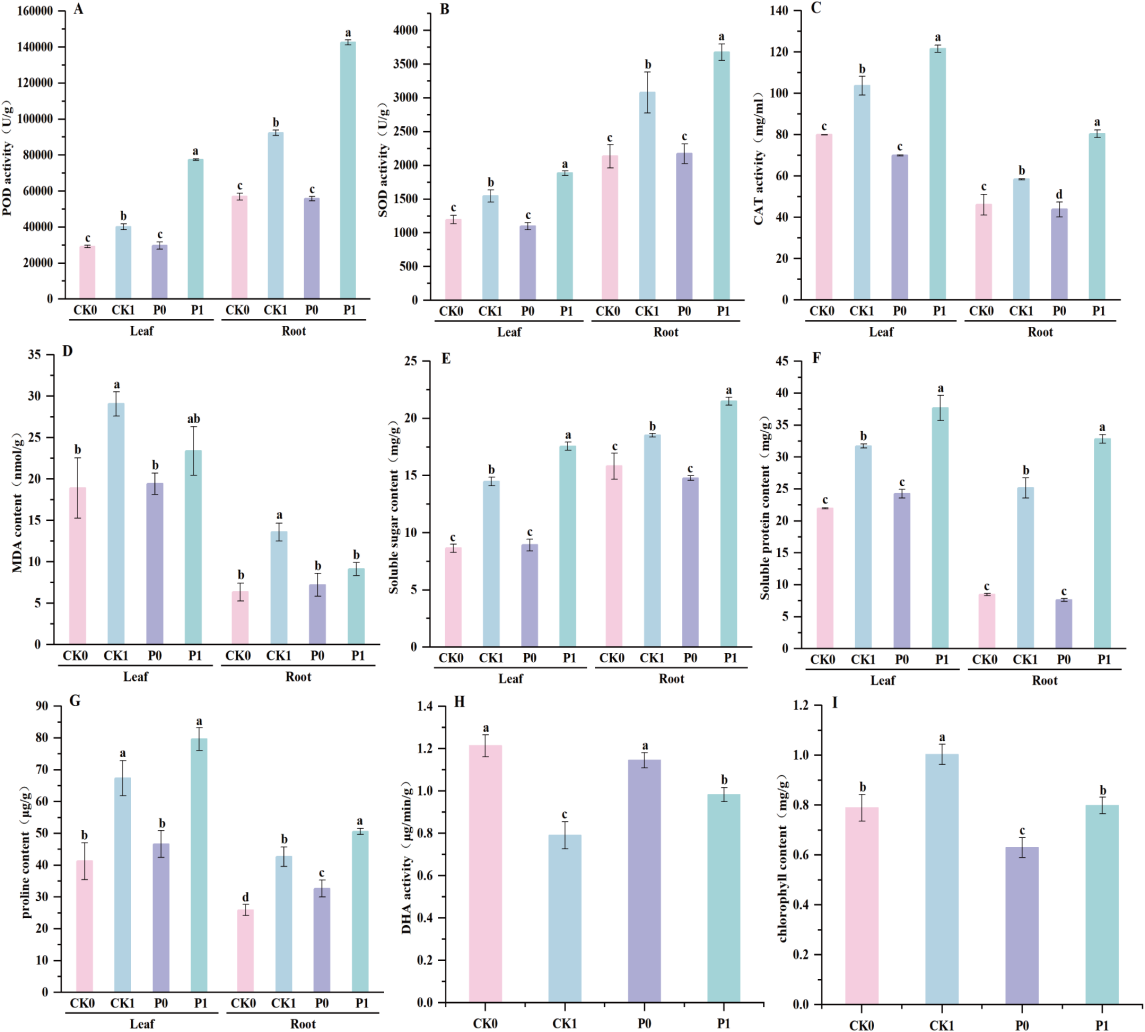
Effects of strain PW2 on POD **(A)**, SOD **(B)**, CAT **(C)**, MDA **(D)**, soluble sugar **(E)**, soluble protein **(F)**, proline **(G)**, DHA **(H)**, and chlorophyll **(I)** of maize seedlings under 100 mmol/L NaCl stress. CK0, control without salt stress; CK1, control under 100 mmol/L salt stress; P0, inoculation group without salt stress; P1, inoculation group under salt stress. Error bars represent standard deviation (n=3). Different lowercase letters indicate significant differences among treatments at *p < 0.05*.

Under salt stress, plants typically enhance their antioxidant defense system to mitigate oxidative damage. Compared to normal conditions (CK0), salt stress (CK1) significantly induced oxidative damage, increasing leaf and root MDA contents by 53.64% and 113.51% (*p < 0.01*), while simultaneously triggering increases in antioxidant enzymes (POD, SOD, CAT) to counteract the stress. Under normal conditions, inoculation with strain PW2 (P0) caused no significant changes in these parameters. However, under salt stress, the P1 treatment significantly amplified this defense. Specifically, compared to the CK1 group, leaf POD increased by 92.74% (77434.67 ± 353.34 U/g *vs.* 40173.33 ± 1547.89 U/g, *p < 0.001*), SOD increased by 21.79% (1884.13 ± 34.46 U/g *vs.* 1547.00 ± 88.15 U/g, *p* = 0.001), and CAT increased by 17.28% (121.64 ± 1.79 U/g *vs.* 103.72 ± 4.56 U/g, *p* = 0.003). This enhanced enzymatic capacity effectively alleviated membrane lipid peroxidation, reducing leaf MDA accumulation by 19.54% (23.39 ± 2.89 nmol/g *vs.* 29.07 ± 1.47 nmol/g, *p* = 0.011). A similar protective effect was observed in roots, where P1 inoculation significantly increased POD by 54.31% (142546.67 ± 1398.22 U/g *vs.* 92373.33 ± 1597.36 U/g, *p < 0.001*), SOD by 19.36% (3676.29 ± 123.05 U/g *vs.* 3080.07 ± 304.32 U/g, *p* = 0.008), and CAT by 37.80% (80.46 ± 1.87 U/g *vs.* 58.39 ± 0.22 U/g, *p* = 0.001), leading to a significant reduction in root MDA by 32.89% (9.12 ± 0.79 nmol/g *vs.* 13.59 ± 1.06 nmol/g, *p* = 0.004). Furthermore, the activities of POD and SOD were consistently higher in roots than in leaves, whereas CAT and MDA levels were higher in leaves than in roots (**Figures 6A–D**).

Under osmotic adjustment substances, significant changes were observed primarily under salt stress and inoculation treatments. Soluble sugars, soluble proteins, and proline are important osmotic adjustment substances in plants; they can lower cellular osmotic potential, maintain osmotic balance between the cytoplasm and vacuole, and protect the integrity of the cell membrane structure. Compared with normal conditions (CK0), under salt stress (CK1), the soluble sugar, soluble protein, and proline in leaves increased by 67.51% (14.48 ± 0.37 mg/g *vs.* 8.64 ± 0.34 mg/g, *p* = 0.0002), 44.35% (31.72 ± 0.33 mg/g *vs.* 21.98 ± 0.08 mg/g, *p < 0.0001*), and 63.31% (67.37 ± 5.57 mg/g *vs.* 41.25 ± 5.38 mg/g, *p* = 0.028), respectively, while those in roots increased by 17.06% (18.52 ± 0.16 mg/g *vs.* 15.82 ± 1.00 mg/g, *p* = 0.015), 197.52% (25.16 ± 1.41 mg/g *vs.* 8.46 ± 0.18 mg/g, *p* = 0.0003), and 64.76% (42.66 ± 3.07 mg/g *vs.* 25.89 ± 1.73 mg/g, *p* = 0.006), respectively. Comparing the normal strain inoculation treatment (P0) with normal conditions (CK0), root proline increased by 26.20% (32.68 ± 2.50 mg/g *vs.* 25.89 ± 1.73 mg/g, *p* = 0.324), and leaf soluble protein also showed a significant increase of 10.46% (24.26 ± 0.59 mg/g *vs.* 21.98 ± 0.08 mg/g, *p* = 0.048). Comparing the salt stress strain inoculation treatment (P1) with the salt stress treatment (CK1), the soluble sugar, soluble protein, and proline in leaves increased by 21.34% (17.57 ± 0.37 mg/g *vs.* 14.48 ± 0.37 mg/g, *p* = 0.0001), 18.87% (37.71 ± 1.78 mg/g *vs.* 31.72 ± 0.33 mg/g, *p* = 0.002), and 18.24% (79.65 ± 3.45 mg/g *vs.* 67.37 ± 5.57 mg/g, *p* = 0.019), respectively, while those in roots increased by 16.09% (21.50 ± 0.34 mg/g *vs.* 18.52 ± 0.16 mg/g, *p* = 0.0001), 30.45% (32.83 ± 0.70 mg/g *vs.* 25.16 ± 1.41 mg/g, *p* = 0.0007), and 18.60% (50.60 ± 0.96 mg/g *vs.* 42.66 ± 3.07 mg/g, *p* = 0.011), respectively. The content of soluble sugar was higher in roots than in leaves, whereas the contents of soluble protein and proline were higher in leaves than in roots (**Figures 6E–G**).

Under osmotic adjustment substances, significant changes were observed primarily under salt stress and inoculation conditions. Dehydrogenase (DHA) and chlorophyll are key indicators reflecting plant metabolic levels. Dehydrogenase can regulate the respiration intensity of the root system, while chlorophyll determines the photosynthetic potential of leaves. Compared with normal conditions (CK0), under salt stress (CK1), DHA decreased by 53.56% (0.791 ± 0.065 g/g *vs.* 1.213 ± 0.051 g/g, *p < 0.0001*) and chlorophyll increased by 27.06% (1.003 ± 0.041 mg/g *vs.* 0.789 ± 0.053 mg/g, *p* = 0.004). Compared with normal conditions (CK0), the strain inoculation treatment under normal conditions (P0) showed no significant change in DHA (1.146 ± 0.036 g/g *vs.* 1.213 ± 0.051 g/g, *p* = 0.132), while chlorophyll decreased by 25.37% (0.630 ± 0.039 mg/g *vs.* 0.789 ± 0.053 mg/g, *p* = 0.014). Compared with the salt stress (CK1), the strain inoculation treatment under salt stress (P1) showed an increase of 24.28% in DHA (0.983 ± 0.033 g/g *vs.* 0.791 ± 0.065 g/g, *p* = 0.009) and a decrease of 25.57% in chlorophyll (0.799 ± 0.033 mg/g *vs.* 1.003 ± 0.041 mg/g, *p* = 0.002) (**Figures 6H, I**).

### 3.8 Multivariate statistical analysis of physiological indicators in maize leaves and roots

**Figure 7:**
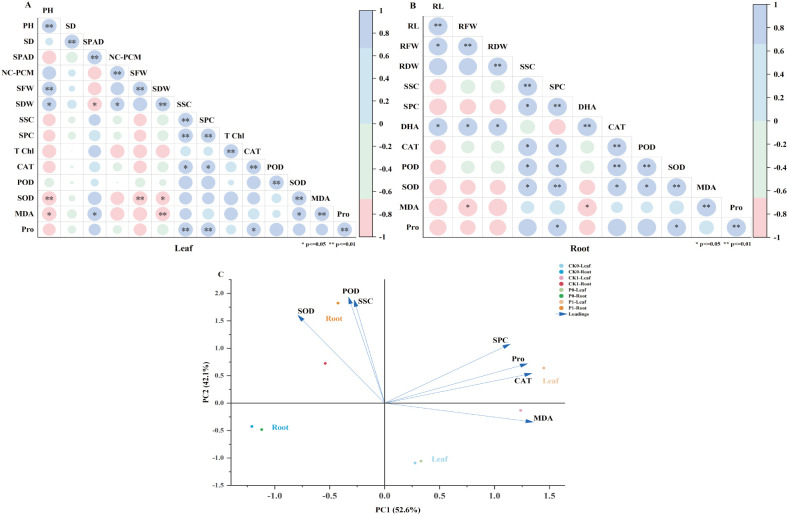
Correlation analysis and principal component analysis of physiological indicators in maize leaves and roots. **(A)** Correlation heatmap of indicators in maize leaves; **(B)** Correlation heatmap of indicators in maize roots.The color gradient from red to blue represents the Pearson correlation coefficient.Red indicates negative correlation, blue indicates positive correlation.*p < 0.05, **p < 0.01 indicate significant correlations. **(C)** PCA biplot of physiological and agronomic indicators in maize leaves and roots.PC1 and PC2 explained 52.6% and 42.1% of the total variance, respectively.

Multivariate statistical analysis was conducted to investigate the relationships among physiological indicators in maize leaves and roots. Correlation analysis and principal component analysis (PCA) results are presented in **Figure 7**.

#### 3.8.1 Correlation analysis

##### 3.8.1.1 Leaf correlation analysis

Highly significant positive correlations (*p < 0.01*) were observed among the antioxidant enzymes SOD, POD, and CAT. These enzymes exhibited highly significant negative correlations (*p < 0.01*) with MDA content. Additionally, a significant positive correlation (*p < 0.05*) was detected between the photosynthetic parameters SPAD and TChl (**Figure 7A**).

##### 3.8.1.2 Root correlation analysis

In the root system, significant positive correlations (*p < 0.05*) were identified between the antioxidant enzymes POD and SOD and the osmolytes DHA and Pro. Highly significant negative correlations (*p < 0.01*) were found between these antioxidant enzymes and MDA content. Furthermore, soluble protein content (SPC) showed significant correlations (*p < 0.05*) with all evaluated physiological indicators (**Figure 7B**).

##### 3.8.1.3 Principal component analysis

The PCA results (**Figure 7C**) revealed that the first two principal components (PC1 and PC2) explained 52.6% and 42.1% of the total variance, respectively, with a cumulative contribution rate of 94.7%. A clear spatial separation was achieved between leaf and root samples along the PC1 axis. Leaf samples predominantly distributed in the positive direction of PC1, closely associated with SPC, Pro, and CAT, whereas root samples clustered in the negative direction, mainly correlating with SOD, POD, and MDA. The distribution of indicator arrows further demonstrated that SSC, POD, and SOD were key drivers for root sample differentiation, while SPC, Pro, and MDA were primary contributors to leaf sample differences. This spatial segregation confirms a significant divergence in physiological metabolic patterns between above-ground and below-ground organs of maize.

## 4 Discussion

This research focused on the screening of salt-tolerant growth-promoting bacteria and the analysis of their mode of action in improving maize seedling resilience under salt stress. From eight effective isolates, a strain exhibiting optimal performance was chosen to determine its plant growth-promoting traits, including nitrogen fixation, mineral solubilization (P and K), IAA production, and ACC deaminase activity (**Table 2**). Additionally, the study evaluated the influence of the strain on growth indices (e.g., biomass, height) (**Figure 5**) and root system configuration (e.g., total root length, surface area) (**Figure 5**, **Table 5**), and physiological and biochemical indices (antioxidant enzymes, osmotic adjustment substances, dehydrogenase, and chlorophyll) of maize seedlings (**Figure 6**), followed by a Multivariate statistical analysis (**Figure 7**). Through these indicators, it was preliminarily confirmed that this strain possesses salt-tolerant growth-promoting properties for maize (Oubaha et al., 2024; Alberghini et al., 2025; Xu et al., 2025). Reports on the plant growth-promoting performance of this specific strain are limited. For instance, Enterobacter asburiae BY4 has been reported to significantly increase the plant height, biomass, leaf area, chlorophyll content, and photosynthetic rate of sugarcane, while activating various defense-related enzyme systems and gene expression (Singh et al., 2021). Under salt stress, inoculation with Enterobacter asburiae D2 significantly improved the growth indices of rice seedlings, including plant height, root length, and root and shoot biomass, and effectively restored chlorophyll content, increased proline accumulation, and reduced malondialdehyde (MDA) content. These findings indicate that the strain promotes growth by enhancing photosynthesis and alleviating osmotic stress and oxidative damage (Ning et al., 2024a). However, to date, there have been no reports on the growth-promoting effects of Enterobacter asburiae on maize or its mechanisms of stress resistance under salt stress. Furthermore, genetic verification is required to determine whether the strain used in this study is identical to those previously reported.

Of all developmental stages, seed germination is the period most vulnerable to the adverse effects of salt stress (Hudu et al., 2025); the vulnerability of its physiological metabolism directly dictates the formation of the plant’s subsequent stress resistance. When seeds are subjected to salt stress, the development of the plumule, radicle, and other embryonic structures is impaired, which subsequently hinders seedling growth and leads to physiological and morphological changes in the plant (Guo et al., 2024). Application of Enterobacter asburiae under salt stress resulted in elevated germination metrics (rate, potential, index, and vigor index) and increased morphological attributes (shoot and root length, fresh and dry weight) of maize (**Table 4**). Consequently, these results indicate that this bacterial strain has the potential to ameliorate the adverse impacts of salt stress on maize growth.

Inoculation of salt-tolerant beneficial bacteria in salt-stressed soil can increase plant biomass while simultaneously alleviating the detrimental effects of salt stress on plants (Akram et al., 2016; Islam et al., 2016; Hmaeid et al., 2019). The results of this study indicate that inoculation with Enterobacter asburiae PW2 significantly improved maize growth under salt stress. The root system serves as the sole connection between the plant’s aerial and underground parts, responsible for transporting water and nutrients to the shoots; furthermore, it is the first organ to be exposed to salt stress and is the most sensitive to changes in soil salinity (Jhuma et al., 2021). The results of this study showed that root morphological indices changed significantly under salt stress; specifically, total root length, number of root tips, root surface area, root volume, and number of root branches were all significantly reduced (**Table 5**). However, inoculation with strain PW2 promoted these root morphological parameters under salt stress to varying degrees.

Salt stress inhibits plant growth and physiological activities by inducing the excessive accumulation of reactive oxygen species (ROS), which leads to membrane lipid peroxidation and structural damage to proteins, ultimately reducing plant biomass and yield. Malondialdehyde (MDA), a key product of membrane lipid peroxidation, serves as a sensitive indicator of the extent of cellular membrane damage (Chen et al., 2025). Under adverse conditions, crops accumulate osmotic adjustment substances to maintain cellular osmotic balance and enhance water retention capacity, thereby facilitating adaptation to the stressful environment. Specifically, soluble proteins significantly enhance cellular water absorption due to their strong hydrophilicity; soluble sugars protect cell membrane and protoplast structures; and free proline effectively maintains tissue water content and prevents cellular dehydration through its excellent water solubility and osmotic regulation functions. Furthermore, root dehydrogenase (DHA) activity serves as an indicator for assessing stress injury, while chlorophyll content directly influences photosynthetic efficiency; both are of significant indicative value for crop responses to stress (Hidri et al., 2016; Lin et al., 2025; Liu et al., 2025). The results of this study indicate that inoculation with strain PW2, compared to the salt-stressed group, increased the activities of CAT, POD, and SOD in both the roots and leaves of maize seedlings, significantly reduced MDA content, and significantly elevated the levels of soluble sugars, soluble proteins, and proline in the roots and leaves. Notably, under salt stress, total chlorophyll content was higher than that in the normal group; however, chlorophyll content decreased following inoculation with strain PW2. This finding contrasts with the majority of studies, which typically report increased chlorophyll content following inoculation. We attribute this discrepancy to a concentration versus dilution effect. Under salt stress, leaf expansion is inhibited, resulting in smaller leaves where chlorophyll becomes relatively concentrated. Conversely, maize seedlings inoculated with strain PW2 exhibited vigorous growth and significant biomass accumulation. This rapid expansion of leaf tissue likely diluted the chlorophyll content per unit fresh weight, resulting in a lower measured value despite the overall improvement in plant growth (Heidari et al., 2014; Du et al., 2025). Regarding proline, previous studies have demonstrated that bioinoculant application can reduce proline accumulation in maize plants under salt stress while activating the antioxidant enzyme defense system (Zaki et al., 2025). In the present study, however, the strain did not significantly decrease proline content in leaves under salt stress, which may be attributed to the persistence of the stress. In roots, salt stress (CK1) increased proline content; interestingly, the P0 treatment (without salt but inoculated with the strain) exhibited significantly higher proline levels than CK0. This may be because PW2, as a salt-tolerant plant growth-promoting bacterium, induces basal osmotic adjustment or promotes root metabolism (e.g., enhancing root vitality and antioxidant capacity) (Lu et al., 2024) under normal growth conditions, thereby leading to proline accumulation. Furthermore, the proline content in the P1 treatment (salt plus strain) was further elevated, indicating that the strain enhanced the osmotic adjustment capacity of the roots to cope with salt stress (Ning et al., 2024a).

## 5 Conclusion

The present study demonstrates that strain PW2 possesses nitrogen fixation, potassium solubilization, and siderophore production capabilities, alongside plant growth-promoting traits including IAA synthesis and ACC deaminase activity. The strain exhibited growth tolerance under NaCl concentrations of 2%–8% and a pH range of 8.0–11.0, and was identified as *Enterobacter asburiae* through physiological, biochemical, and 16S rRNA gene sequencing analyses. Under salt stress, inoculation with PW2 significantly improved the growth performance of maize seedlings; specifically, shoot biomass and root length increased by 47.83% and 36.72%, respectively, compared to non-inoculated controls. Furthermore, the strain enhanced seed germination, root vitality, osmotic adjustment, and antioxidant defenses. Based on these pot experiments, PW2 shows promising potential as a bioinoculant for maize cultivation in saline-alkali soils, offering a sustainable alternative to chemical fertilizers. However, further field trials are warranted to validate the practical growth-promoting efficacy and colonization ability of this strain under complex field conditions, thereby providing support for its commercial application.

## Data Availability

The original contributions presented in the study are included in the article. The 16S rRNA gene sequence of *Enterobacter asburiae* strain PW2 has been deposited in the NCBI GenBank database under the accession number PZ245473.1.

## References

[B1] Akram M. S. Shahid M. Tariq M. Azeem M. Javed M. T. Saleem S. . (2016). Deciphering staphylococcus sciuri SAT-17 mediated anti-oxidative defense mechanisms and growth modulations in salt stressed maize (zea mays L.). Front. Microbiol. 7. doi: 10.3389/fmicb.2016.00867. PMID: 27375588 PMC4899454

[B2] Al-Turki A. Murali M. Omar A. F. Rehan M. Sayyed R. Z. (2023). Recent advances in PGPR-mediated resilience toward interactive effects of drought and salt stress in plants. Front. Microbiol. 14, 1214845. doi: 10.3389/fmicb.2023.1214845. PMID: 37829451 PMC10565232

[B3] Alberghini B. Vicino M. Zanetti F. Silvestre S. Haslam R. Zegada-Lizarazu W. . (2025). Assessing different physiological, seed yield and quality responses of camelina lines to drought. Ind. Crops Prod. 234, 121528. doi: 10.1016/j.indcrop.2025.121528. PMID: 38826717

[B4] Chen Z. Zhang P. Wang B. Li H. Li S. Zhang H. . (2025). Harnessing the role of rhizo-bacteria to mitigate salinity stress in rice (orzya sativa); focus on antioxidant defense system, photosynthesis response, and rhizosphere microbial diversity. Rhizosphere 33, 101043. doi: 10.1016/j.rhisph.2025.101043. PMID: 38826717

[B5] Du X.-L. Feng N.-J. Zheng D.-F. Lin Y. Zhou H. Li J.-H. . (2025). Effects of exogenous uniconazole (S3307) on oxidative damage and carbon metabolism of rice under salt stress. BMC Plant Biol. 25, 541. doi: 10.1186/s12870-025-06467-0. PMID: 40281403 PMC12032716

[B6] Fu Y. Zheng G. Ma L. Li J. Hou D. Zhang L. . (2025). Metabolite accumulation contributes to differences in seed germination of water-saving and drought-resistance rice under dry direct seeding. BMC Plant Biol. 25, 1417. doi: 10.1186/s12870-025-07405-w. PMID: 41120893 PMC12538913

[B7] Glick B. R. (2012). Plant growth-promoting bacteria: Mechanisms and applications. Scientifica 2012, 1–15. doi: 10.6064/2012/963401. PMID: 24278762 PMC3820493

[B8] Glickmann E. Dessaux Y. (1995). A critical examination of the specificity of the salkowski reagent for indolic compounds produced by phytopathogenic bacteria. Appl. Environ. Microbiol. 61, 793–796. doi: 10.1128/aem.61.2.793-796.1995. PMID: 16534942 PMC1388360

[B9] Guo M. Zong J. Zhang J. Wei L. Wei W. Fan R. . (2024). Effects of temperature and drought stress on the seed germination of a peatland lily (lilium concolor var. megalanthum). Front. Plant Sci. 15, 1462655. doi: 10.3389/fpls.2024.1462655. PMID: 39469053 PMC11514071

[B10] Heidari A. Bandehagh A. Toorchi M. (2014). Effects of NaCl stress on chlorophyll content and chlorophyll fluorescence in sunflower (helianthus annuus L.) lines. Yüzüncü Yıl Üniversitesi Tarım Bilimleri Dergisi 24, 111–120. doi: 10.29133/yyutbd.235924

[B11] Hidri R. Barea J. M. Mahmoud O. M.-B. Abdelly C. Azcón R. (2016). Impact of microbial inoculation on biomass accumulation by sulla carnosa provenances, and in regulating nutrition, physiological and antioxidant activities of this species under non-saline and saline conditions. J. Plant Physiol. 201, 28–41. doi: 10.1016/j.jplph.2016.06.013. PMID: 27393918

[B12] Hmaeid N. Wali M. Metoui-Ben Mahmoud O. Pueyo J. J. Ghnaya T. Abdelly C. (2019). Efficient rhizobacteria promote growth and alleviate NaCl-induced stress in the plant species sulla carnosa. Appl. Soil Ecol. 133, 104–113. doi: 10.1016/j.apsoil.2018.09.011. PMID: 38826717

[B13] Holt J. G. Krieg N. R. Sneath P. H. A. Staley J. T. (1994). Bergey’s Manual of Determinative Bacteriology Vol. 9th ed (Baltimore, MD: Williams & Wilkins).

[B14] Hudu A. R. Kankam F. Nanmang I. A. Dawuda M. M. Hamid H. H. Opoku N. (2025). Identification of fusarium verticillioides isolates and their impact on seed germination and biochemical profiles in maize. Plant Enviro Interact. 6, e70104. doi: 10.1002/pei3.70104. PMID: 41446435 PMC12724013

[B15] Iqbal M. Z. Singh K. Chandra R. (2024). Recent advances of plant growth promoting rhizobacteria (PGPR) for eco-restoration of polluted soil. Cleaner Eng. Technol. 23, 100845. doi: 10.1016/j.clet.2024.100845. PMID: 38826717

[B16] Islam F. Yasmeen T. Arif M. S. Ali S. Ali B. Hameed S. . (2016). Plant growth promoting bacteria confer salt tolerance in vigna radiata by up-regulating antioxidant defense and biological soil fertility. Plant Growth Regul. 80, 23–36. doi: 10.1007/s10725-015-0142-y. PMID: 30311153

[B17] Jhuma T. A. Rafeya J. Sultana S. Rahman M. T. Karim M. M. (2021). Isolation of endophytic salt-tolerant plant growth-promoting rhizobacteria from oryza sativa and evaluation of their plant growth-promoting traits under salinity stress condition. Front. Sustain. Food. Syst. 5, 687531. doi: 10.3389/fsufs.2021.687531

[B18] Kubi H. A. A. Khan M. A. Adhikari A. Imran M. Kang S.-M. Hamayun M. . (2021). Silicon and Plant Growth-Promoting Rhizobacteria Pseudomonas psychrotolerans CS51 Mitigates Salt Stress in Zea mays L. Agriculture 11, 272. doi: 10.3390/agriculture11030272. PMID: 30654563

[B19] Kumar A. Singh S. Mukherjee A. Rastogi R. P. Verma J. P. (2021). Salt-tolerant plant growth-promoting bacillus pumilus strain JPVS11 to enhance plant growth attributes of rice and improve soil health under salinity stress. Microbiol. Res. 242, 126616. doi: 10.1016/j.micres.2020.126616. PMID: 33115624

[B20] Li P. Liu Q. Wei Y. Xing C. Xu Z. Ding F. . (2024). Transcriptional landscape of cotton roots in response to salt stress at single-cell resolution. Plant Commun. 5, 100740. doi: 10.1016/j.xplc.2023.100740. PMID: 39492159 PMC10873896

[B21] Lin T. Haider F. U. Liu T. Li S. Zhang P. Zhao C. . (2025). Salt tolerance induced by plant growth-promoting rhizobacteria is associated with modulations of the photosynthetic characteristics, antioxidant system, and rhizosphere microbial diversity in soybean (glycine max (L.) merr.). Agronomy 15, 341. doi: 10.3390/agronomy15020341. PMID: 30654563

[B22] Liu J. Zhao X. Niu Y. Ren Y. Wang M. Han B. . (2025). Plant growth-promoting rhizobacteria halomonas alkaliAntarcticae M23 promotes the salt tolerance of maize by increasing the K+/na+ ratio, antioxidant levels, and ABA levels and changing the rhizosphere bacterial community. BMC Plant Biol. 25, 727. doi: 10.1186/s12870-025-06765-7. PMID: 40442582 PMC12121225

[B23] Lu L. Liu N. Fan Z. Liu M. Zhang X. Tian J. . (2024). A novel PGPR strain, streptomyces lasalocidi JCM 3373^T^, alleviates salt stress and shapes root architecture in soybean by secreting indole‐3‐carboxaldehyde. Plant Cell Environ. 47, 1941–1956. doi: 10.1111/pce.14847. PMID: 38369767

[B24] Maimaiti A. Gu W. Yu D. Guan Y. Qu J. Qin T. . (2025). Dynamic molecular regulation of salt stress responses in maize (zea mays L.) seedlings. Front. Plant Sci. 16, 1535943. doi: 10.3389/fpls.2025.1535943. PMID: 40070712 PMC11893837

[B25] Mishra P. Mishra J. Arora N. K. (2021). Plant growth promoting bacteria for combating salinity stress in plants – recent developments and prospects: A review. Microbiol. Res. 252, 126861. doi: 10.1016/j.micres.2021.126861. PMID: 34521049

[B26] Ning Z. Lin K. Gao M. Han X. Guan Q. Ji X. . (2024a). Mitigation of salt stress in rice by the halotolerant plant growth-promoting bacterium enterobacter asburiae D2. JoX 14, 333–349. doi: 10.3390/jox14010021. PMID: 38535496 PMC10971743

[B27] Oubaha B. Rathore R. S. Bagri J. Singhal N. K. Mazumdar K. Rishi V. . (2024). Bacillus siamensis strain BW enhances rice growth and salinity tolerance through redox equilibrium and hormone modulation. Curr. Plant Biol. 37, 100321. doi: 10.1016/j.cpb.2024.100321. PMID: 38826717

[B28] Penrose D. M. Glick B. R. (2003). Methods for isolating and characterizing ACC deaminase‐containing plant growth‐promoting rhizobacteria. Physiol. Plant 118, 10–15. doi: 10.1034/j.1399-3054.2003.00086.x. PMID: 12702008

[B29] Prerna D. I. Govindaraju K. Tamilselvan S. Kannan M. Vasantharaja R. Chaturvedi S. . (2021). Influence of nanoscale micro-nutrient α-Fe2O3 on seed germination, seedling growth, translocation, physiological effects and yield of rice (oryza sativa) and maize (zea mays). Plant Physiol. Biochem. 162, 564–580. doi: 10.1016/j.plaphy.2021.03.023. PMID: 33773232

[B30] Radhakrishnan R. Hashem A. Abd_Allah E. F. (2017). Bacillus: A biological tool for crop improvement through bio-molecular changes in adverse environments. Front. Physiol. 8, 667. doi: 10.3389/fphys.2017.00667. PMID: 28932199 PMC5592640

[B31] Rahbari K. Madandoust M. (2024). The effect of seed moisture content of hybrid maize at harvest time on seed germination traits and antioxidant enzymes activity under simulated environmental stresses with silicon foliar application. Silicon 16, 3629–3639. doi: 10.1007/s12633-024-02953-6. PMID: 30311153

[B32] Schwyn B. Neilands J. B. (1987). Universal chemical assay for the detection and determination of siderophores. Anal. Biochem. 160, 47–56. doi: 10.1016/0003-2697(87)90612-9. PMID: 2952030

[B33] Siddika A. Rashid A. A. Khan S. N. Khatun A. Karim M. M. Prasad P. V. V. . (2024). Harnessing plant growth-promoting rhizobacteria, bacillus subtilis and B. aryabhattai to combat salt stress in rice: A study on the regulation of antioxidant defense, ion homeostasis, and photosynthetic parameters. Front. Plant Sci. 15, 1419764. doi: 10.3389/fpls.2024.1419764. PMID: 38938633 PMC11208634

[B34] Singh P. Singh R. K. Li H.-B. Guo D.-J. Sharma A. Lakshmanan P. . (2021). Diazotrophic bacteria pantoea dispersa and enterobacter asburiae promote sugarcane growth by inducing nitrogen uptake and defense-related gene expression. Front. Microbiol. 11, 600417. doi: 10.3389/fmicb.2020.600417. PMID: 33510724 PMC7835727

[B35] Wang C. Wei X. Wang Y. Wu C. Jiao P. Jiang Z. . (2025). Metabolomics and transcriptomic analysis revealed the response mechanism of maize to saline‐Alkali stress. Plant Biotechnol. J. 23, 5397–5416. doi: 10.1111/pbi.70292. PMID: 40815574 PMC12665074

[B36] Wang Y. Liang C. Meng Z. Li Y. Abid M. A. Askari M. . (2019). Leveraging Atriplex hortensis choline monooxygenase to improve chilling tolerance in cotton. Environ. Exp. Bot. 162, 364–373. doi: 10.1016/j.envexpbot.2019.03.012. PMID: 38826717

[B37] Wang Y. Pei Y. Wang X. Dai X. Zhu M. (2024). Antimicrobial metabolites produced by the plant growth-promoting rhizobacteria (PGPR): Bacillus and pseudomonas. Adv. Agrochem 3, 206–221. doi: 10.1016/j.aac.2024.07.007. PMID: 38826717

[B38] Xiao S. Song W. Xing J. Su A. Zhao Y. Li C. . (2023). ORF355 confers enhanced salinity stress adaptability to S‐type cytoplasmic male sterility maize by modulating the mitochondrial metabolic homeostasis. JIPB 65, 656–673. doi: 10.1111/jipb.13382. PMID: 36223073

[B39] Xu Y. Qiu L. Zhuang W. Wang Z. Wang T. Xie Y. (2025). Effects of melatonin on growth, photosynthesis, and physiological characteristics in Pseudostellaria heterophylla during the late growth stage. Ind. Crops Prod. 236, 121992. doi: 10.1016/j.indcrop.2025.121992. PMID: 38826717

[B40] Yang D. Tang L. Chen J. Shi Y. Zhou H. Gao H. . (2024). Strategy of endophytic bacterial communities in alfalfa roots for enhancing plant resilience to saline–alkali stress and its application. Biol. Fertil. Soils 60, 493–507. doi: 10.1007/s00374-024-01816-x. PMID: 30311153

[B41] Yin Y.-J. Chen C.-J. Guo S.-W. Li K.-M. Ma Y.-N. Sun W.-M. . (2018). The fight against panax notoginseng root-rot disease using zingiberaceae essential oils as potential weapons. Front. Plant Sci. 9, 1346. doi: 10.3389/fpls.2018.01346. PMID: 30337932 PMC6180181

[B42] Zakavi M. Askari H. Shahrooei M. (2022). Maize growth response to different bacillus strains isolated from a salt-marshland area under salinity stress. BMC Plant Biol. 22, 367. doi: 10.1186/s12870-022-03702-w. PMID: 35879654 PMC9317119

[B43] Zaki R. M. Afify A. H. Ashour E. H. El-Sawah A. M. (2025). Salt-tolerant bacteria support salinity stress mitigating impact of arbuscular mycorrhizal fungi in maize (zea mays L.). Microorganisms 13, 1345. doi: 10.3390/microorganisms13061345. PMID: 40572232 PMC12196076

