# Supplementary material for: Promotion of maize seedling growth by Enterobacter asburiae PW2 under salt stress
Source: Front Plant Sci. 2026 May 4;17:1823368. doi: 10.3389/fpls.2026.1823368 (PMC13180849; doi:10.3389/fpls.2026.1823368)
Supplement: Supplementary file 1 [file DataSheet1.pdf]

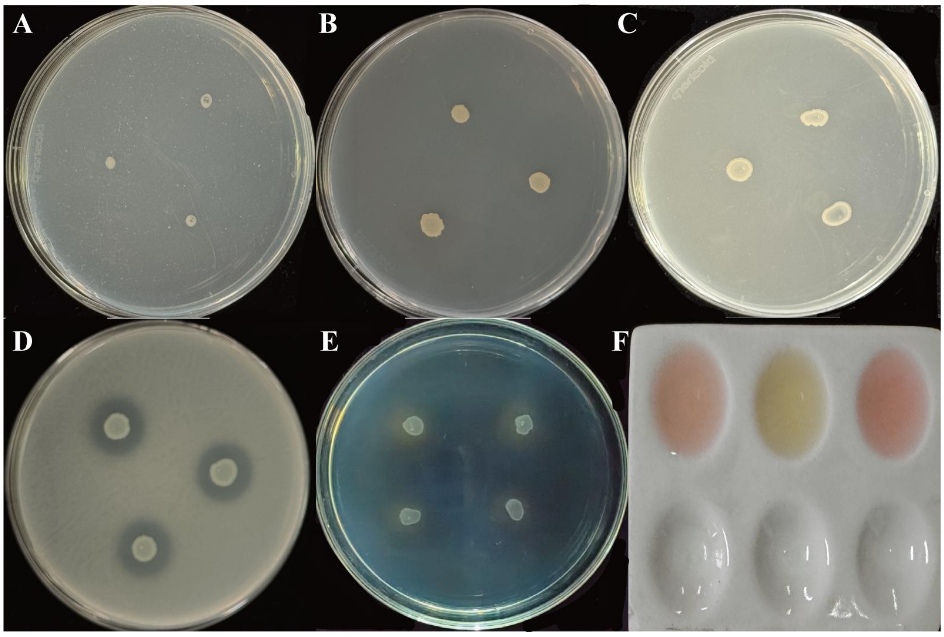

Figure S1. Promotional performance of strain PW2

(A): Nitrogen fixation; (B): Organophosphate solubilization; (C): Inorganic phosphate solubilization; (D): Potassium solubilization; (E): Siderophore production; (F): IAA synthesis (From left to right: positive control, negative control, and *Enterobacter asburiae* strain)

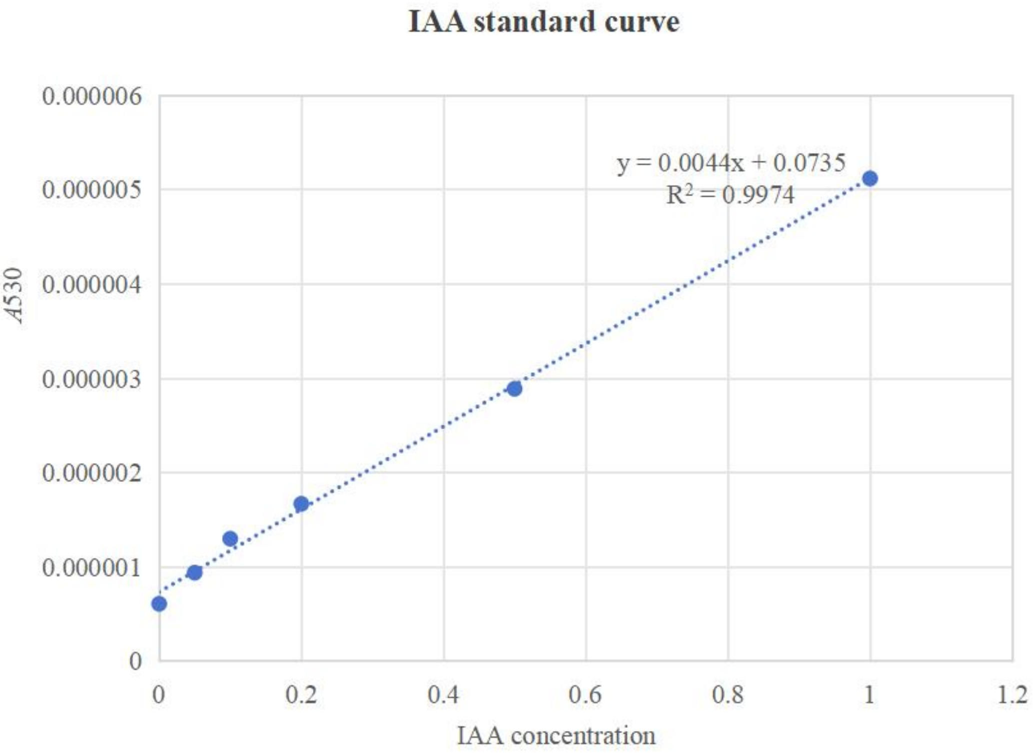

Figure S2. Quantification of IAA production by *Enterobacter asburiae* strain
